# Where do nursing students make mistakes when calculating drug doses? A retrospective study

**DOI:** 10.1186/s12912-022-01085-9

**Published:** 2022-11-10

**Authors:** Laia Wennberg-Capellades, Pilar Fuster-Linares, Encarnación Rodríguez-Higueras, Alberto Gallart Fernández-Puebla, Mireia Llaurado-Serra

**Affiliations:** Department of Nursing, School of Medicine and Health Sciences, Universitat Internacional de Catalunya, Josep Trueta s/n, 08195 Barcelona, Sant Cugat del Vallès Spain

**Keywords:** Dosage calculation, Nurse education, Medication errors, Nursing students, Patient safety

## Abstract

**Background:**

Research internationally shows that nursing students find dosage calculation difficult. Identifying the specific aspects of dose calculation procedures that are most commonly associated with errors would enable teaching to be targeted where it is most needed, thus improving students’ calculation skills. The aim of this study was to analyze where specifically nursing students make mistakes when calculating drug doses.

**Method:**

Retrospective analysis of written examination papers including dosage calculation exercises from years 1, 2, and 3 of a nursing degree program. Exercises were analyzed for errors in relation to 23 agreed categories reflecting different kinds of calculation or steps in the calculation process. We conducted a descriptive and bivariate analysis of results, examining the relationship between the presence of errors and the proportion of correct and incorrect final answers.

**Results:**

A total of 285 exam papers including 1034 calculation exercises were reviewed. After excluding those that had been left blank, a total of 863 exercises were analyzed in detail. A correct answer was given in 455 exercises (52.7%), although this varied enormously depending on the type of exercise: 89.2% of basic dose calculations were correct, compared with just 2.9% of those involving consideration of maximum concentration. The most common errors were related to unit conversion, more complex concepts such as maximum concentration and minimum dilution, or failure to contextualize the answer to the clinical case. Other frequent errors involved not extracting the key information from the question, not including the units when giving their answer, and not understanding the question. In general, fewer errors in basic dose calculations were made by students at later stages of the degree program.

**Conclusions:**

Students struggle with more complex dose calculations. The main errors detected were related to understanding the task and the key concepts involved, as well as not following the correct steps when solving the problem.

**Supplementary Information:**

The online version contains supplementary material available at 10.1186/s12912-022-01085-9.

## Background

Dosage calculation errors can have serious consequences when administering medicines, and hence numeracy skills are crucial for ensuring safe medication management [1]. A study by Ross et al. [2] in a pediatric teaching hospital found that 8% of medication incidents involved a tenfold error in the dose administered. More recently, a systematic review of intravenous admixture drug preparation errors found that the reported incidence of wrong doses across the studies reviewed ranged from 0 to 32.6%, while for wrong diluent volume the range was 0.06 to 49.0%. The authors highlighted the need in future research to develop standardized definitions for these types of errors so as to facilitate a better understanding of where they happen within the drug preparation process and to devise ways of avoiding them [3]. This underlines an issue raised by other authors, namely that it is difficult to know whether an error in the final dose administered is due solely or primarily to miscalculation, insofar as it may be the result of an error further back in the process, such as pharmacy mislabeling or incorrect or unclear prescribing [2].

Research focusing on nursing students suggests they often have poor drug calculation skills, due especially to difficulties with understanding mathematical principles [4]. Accordingly, although they are usually able to perform simple calculations, they struggle with tasks involving multiple steps and which require a higher level of conceptual knowledge [5]. A further issue concerns the extent to which nursing students are aware of their errors. In this regard, a recent study in which students were asked to indicate their level of certainty about answers given to a pharmacology knowledge questionnaire concluded that there was a high risk of medication administration error in 14% of the students who rated incorrect answers with high certainty [6].

Aside from their arithmetic skills, there are a number of other factors that may contribute to nursing students’ difficulties with dose calculation. One of these is math anxiety, which can undermine their ability to understand and complete tasks involving mathematics [7]. A fear of math, resulting in resistance to learning math for medication administration, has also been noted in research using focus groups to explore students’ own perspectives on learning math for medication calculation [8]. Other themes that emerged in the same study were: resentment among students towards what they perceive as ‘complicated’ math; lack of confidence among students leading to a fear of error in clinical practice; a recognition among students that they need to be more self-directed in developing their math skills; and the need for clinical instructors to be consistent in giving students the opportunity to practice calculations in the clinical setting [8].

Notwithstanding the difficulties that students experience with dose calculation, the literature suggests that the problem is far from unsurmountable. Indeed, various studies have reported the effectiveness of workshops or web-based platforms designed to support students’ learning and improve their medication calculation skills [9–13].

Another issue to consider, and one highlighted in a recent comparative study of six European countries [5], concerns cross-national differences in medication education regulations and practices and the competences that graduating nurses are expected to have acquired. For instance, some professional bodies such as the UK Nursing & Midwifery Council recommend that nurse education institutions should require students to achieve a 100% pass on a health numeracy assessment including calculation of medicines [14]. In our country, Spain, a strict criterion such as this is not applied within nurse education programs, although *safe medication management* is considered a key competence for students to acquire during their pre-registration university training [15].

Whatever the requirements and approach to training, it is vital that nursing students acquire adequate numeracy skills, as once they enter professional practice they will be responsible for administering medication. The importance of their becoming proficient in this respect by the time they graduate is underlined by research suggesting that difficulties with drug calculation often persist among registered nurses [16–18]. From the perspective of nurse education, therefore, it is crucial to identify where students struggle the most when it comes to dosage calculation. Despite this, few studies have examined in detail the specific aspects of the drug calculation process where students make mistakes [7, 19–21].

Given that the dose calculation skills of both student and registered nurses is an issue of international concern, and one that has implications for patient safety, our aim in the present study was to conduct an in-depth analysis of the kinds of errors that undergraduate nursing students make when performing dose calculation exercises. Identifying the specific aspects or steps in the process they find most difficult would enable nurse educators to target teaching where it is most needed.

## Method

### Design

This was a descriptive retrospective study in which we reviewed all the examination papers that included at least one dose calculation exercise and which had been submitted over two academic years (2017-18 and 2018-19) by students from years 1, 2, and 3 of a nursing degree program at our university.

### Setting

In Spain, nursing degrees last 4 years, and successful graduates are eligible to perform independently oral and higher risk medication management, including intravenous injections and infusions [15]. Nursing students at our university begin to be taught dose calculation and medication administration in year one of the degree program. This is done through both theory classes and low-fidelity simulation, totaling 6 hours. At this stage of their training, students learn how to administer medication through different routes (e.g., oral, IV) and to make simple dose calculations on paper (exercises such as “Basic dose calculation” or, “Infusion rate”; see Supplementary material 1 for definitions). In year 2, students attend a 2-hour theory class in which they are required to perform the same exercises as in year 1, as well as new types of calculation such as “Unit conversion”, “Dose according to patient’s weight”, and “Dose calculation involving a percentage”. In year 3, theory classes and low-fidelity simulation (totaling 16 hours) are used to provide students with further practice in the aforementioned types of calculation exercise and different routes of drug administration, this time incorporating more advanced aspects such as the use of infusion pumps. In year 4, students do not receive classroom instruction in dose calculation or medication administration as the year is spent almost entirely on clinical placement.

Students begin clinical placements in year 1 of the degree program, following completion of all the theory classes. Over the 4 years of their studies, they complete a total of 2300 hours on placement. Because students are assigned to different clinical settings, the experience they gain in relation to dose calculation and medication administration may vary.

### Examination papers reviewed

All the examinations reviewed were time limited and had been sat in the presence of an invigilator, subsequent to having received the instruction corresponding to each course year (see Setting). For year 1 students, the dose calculation exercise formed part of a written station of an objective structured clinical examination (OSCE). The calculation exercises in year 2 were part of a written exam and consisted of 10 questions of varying difficulty and complexity. This part of the exam had been purposely designed to include some calculation exercises that went beyond the level required of year 2 students, and thus they would not necessarily be expected to answer them all correctly (exercises 7, 8, and 9 in the Year 2 block of Table 1). The rationale for including more difficult problems was that we would then be able to track the progress of individual students by setting them the same questions (changing only the numerical values involved) in years 3 and 4 of their studies (the questionnaire used is shown in Supplementary material 2). This progress monitoring is not part of the present analysis. Finally, the exercises corresponding to year 3 formed part of an ordinary written exam and of an OSCE. Table 1 summarizes the exercises used in each of the 3 years; further details, with examples, are given in Supplementary material 3.

**Table 1:** Types of exercises included in the analysis

Year 1	Clinical case of an adult patient in which students must calculate the dose of a prescribed drug from the stock available. They also have to calculate the rate at which the drug should be administered.
Year 2	Written exam comprising 10 calculation exercises, as follows: 1) Unit equivalences (theoretical); 2 and 3) basic dose calculations; 4) calculation requiring unit conversion; 5) dose calculation involving a percentage; 6) dose according to patient’s weight; 7) drug concentration over time; 8) total infusion time, taking into account the maximum flow rate; 9) volume of diluent, taking into account the maximum concentration; 10) infusion rate. Exercises 6, 8, and 9 referred to a pediatric patient, while the remainder concerned adults. With the exception of exercise 1, all the questions were formulated using standard technical language.
Year 3	Four exercises distributed across two exams: Clinical case of an adult critical patient: 1) the calculation involves maintaining a prescribed IV dose for a different drug concentration and infusion rate. Clinical pediatric cases: 2) calculation of IV dose based on patient’s weight and with unit conversion, plus calculation of infusion rate; 3) calculation of oral medication based on patient’s weight; 4) calculation of IV dose based on patient’s weight and with unit conversion, plus calculation of infusion rate and concentration.

### Procedure

In a first step, we selected a random sample of papers covering all the different exams. In the absence of an existing rubric for classifying errors, two members of the research team, working independently, then carried out an initial content analysis in order to categorize the different types of error made by students in each exercise. This provisional set of categories was then agreed with the rest of the team and checked for clarity and relevance by asking each team member to apply it to a small sample of exam papers. This process yielded a consensus list of 23 categories that were used to analyze the total sample of papers (see Supplementary material 3). For this final analysis, and given the large number of records, the exam papers were distributed among pairs of researchers, who first reviewed them individually before comparing and discussing their evaluation with that of the co-evaluator so as to reach a consensus decision. The task in each case was to record 1) whether the category was applicable or not to a particular exercise, and 2) whether the student’s answer took into account the aspect referred to by the category, and if so whether they did so correctly, partially or incorrectly. At this stage in the process, papers were coded so that only the evaluator knew the identity of the student. The results of this analysis were recorded using a spreadsheet, which was then reviewed by the principal investigator (PI) to check for any inconsistencies or errors (e.g., the category regarding unit conversion was wrongly recorded as being applicable to an exercise that did not require this calculation); in the event that a problem was identified, the PI asked the evaluator who had reviewed the corresponding exam paper to make the necessary correction(s) to the database. Once the accuracy of the database had been checked, its contents were fully anonymized by deleting the aforementioned codes.

### Data analysis

Any exercises that were left blank and had not been attempted by the student were excluded. For the descriptive analysis we calculated absolute and relative frequencies, and where appropriate the mean and standard deviation. Bivariate analysis using either the chi-square or Fisher’s exact test, as appropriate, was then conducted to examine whether there were significant differences between the proportion of correct and incorrect answers depending on the presence of errors in each of the aspects (categories) analyzed. McNemar’s test was used for the analysis of paired data. All data analyses were performed using SPSS for Windows 21. When categorizing errors, the researchers sometimes added qualitative comments either to clarify the nature of the error or to justify their choice of category. Those comments that clarified the nature of errors or which provided extra information about them were logged and are considered in the presentation of results.

For years 2 and 3, where more than one exercise was analyzed for each student, we calculated the overall mark out of 10 so as to have an overview of the student’s level of knowledge and to facilitate discussion of results. However, we also analyzed the individual results for each exercise.

### Ethical considerations

Approval for the study was granted by both the Department of Nursing and the Research Ethics Committee of the Universitat Internacional de Catalunya (ref. INF-2018-05). The need for informed consent was waived by the ethics committee due to the retrospective nature of the study (see Procedure section). All methods were carried out in accordance with relevant guidelines and regulations.

In preparing the present article, we referred to the STROBE checklist of items that should be included in reports of descriptive retrospective studies [22].

## Results

We reviewed 285 examination papers that included 1034 calculation exercises. After excluding those exercises that had been left blank (*n* = 171), a total of 863 exercises were analyzed. Table 2 shows the number of exercises reviewed and analyzed for each of the three course years.

**Table 2:** Distribution of calculation exercises reviewed and analyzed by course year

Year	Source	N° exercises reviewed	N° (%) exercises analyzed
1	Clinical case (written) of adult patient	95	88 (92.6)
2	Written exam, 10 exercises	690	536 (77.6)
3	Clinical case (written) of adult critical patient	57	50 (87.7)
3	Clinical cases (written) of pediatric patients	192	189 (96.9)
	**TOTAL**	**1034**	**863 (83.5)**

Of the exercises analyzed, 455 (52.7%) were answered correctly. Table 3 shows the number of each type of exercise that were attempted and the number that were correct. Overall, a correct answer was given in 28.4% of the clinical case exercises in year 1, in 50.9% of the exercises in year 2 (mean score of 5.2 (SD 2.2) out of 10), and in 41.8% of those in year 3. The mean score across the three pediatric exercises in year 3 (final three rows in Table 3) was 4.6 (SD 3.2) out of 10.

**Table 3:** Calculation exercises attempted and those answered correctly in the sample analyzed

Year	Type of calculation required by the exercise	Total n exercises reviewed	Exercises attempted, n (%)	Answered correctly, n (%)^a^
1	Dose concentration and the corresponding infusion rate	95	88 (92.6)	27 (28.4)
2	Unit equivalences (theoretical)	69	69 (100)	8 (11.6)^b^
2	Basic dose calculation	138	129 (93.4)	117 (84.2)
2	Unit conversion	69	57 (82.6)	41 (59.4)
2	Total infusion time, taking into account the maximum flow rate	69	53 (76.8)	23 (33.3)
2	Dose calculation involving a percentage	69	53 (76.8)	48 (69.6)
2	Dose according to patient’s weight	69	56 (81.1)	44 (63.8)
2	Drug concentration over time (mg/min)	69	43 (62.3)	31 (44.9)
2	Volume of diluent, taking into account the maximum concentration	69	38 (55.1)	2 (2.9)
2	Infusion rate	69	38 (55.1)	10 (14.5)
3	Maintain prescribed dose for different concentration and infusion rate	57	50 (87.7)	17 (29.8)
3	IV dose based on patient’s weight and with unit conversion, plus calculation of infusion rate	64	64 (100)	21 (32.8)
3	Oral dose based on patient’s weight	64	64 (100)	45 (70.3)
3	IV dose based on patient’s weight and with unit conversion, plus calculation of infusion rate and concentration	64	61 (95.3)	21 (32.8)
**Total**		**1034**	**863 (83.5)**	**455 (52.7)**

^a^The % of correct answers is calculated based on the total number of exercises reviewed, including those left blank (*n* = 1034). ^b^Students who correctly answered all the unit equivalence (theory) questions. All exercises were contextualized, with the exception of unit equivalences

Regarding the method used to solve the calculation problems (not including the 69 exercises corresponding to theoretical unit equivalences), in 67.1% (*n* = 533) of cases the student used the rule-of-three method, in 18.0% (*n* = 143) a conversion factor (i.e. a number for changing given units to desired units), and in 3.1% (*n* = 25) both these methods. In 93 exercises (11.7%), the student gave an answer directly without using either of these methods. Overall, 90% of the exercises analyzed were considered complete as the student indicated a final answer to the problem, although the result was only correct in 52.7% of cases.

### Most common errors

Table 4 shows the different aspects of the calculation exercises where errors were observed, distinguishing between answers that were ultimately correct or incorrect. For each of these aspects or categories, we examined statistically the relationship between the presence of errors and the proportions of correct and incorrect final answers. It can be seen that with the exception of two categories (i.e., consideration of the diluent solution, and error carried forward), the presence of errors was associated with a significantly higher proportion of incorrect final answers. The areas where errors were most frequently observed concerned an understanding of percentages and unit equivalences (51.6 and 37.7%, respectively), as well as calculations involving more advanced concepts such as maximum concentration and minimum dilution (48.7%). We also found, based on those exercises where it could be analyzed, that in around a third of cases (32.9%) students did not check whether their answer made sense and was realistic, with a related problem being failure to contextualize their result to the patient in question (25.3%).

**Table 4:** Aspects of the exercises analyzed where errors were observed

N (%)		Overall	Incorrect final answer	Correct final answer	***p***-value
Uses information given about the stock available (*n* = 705)	No	10 (1.4)	9 (3.2)	1 (0.2)	< .001
	Partially	15 (2.1)	12 (4.2)	3 (0.7)	
	Incorrectly	31 (4.4)	29 (10.2)	2 (0.5)	
	Correctly	649 (92.1)	235 (82.5)	414 (98.6)	
Consideration of the diluent solution (*n* = 206)	No	3 (1.5)	3 (3.0)	0	.075*
	Partial	4 (1.9)	3 (3.0)	1 (0.9)	
	Incorrect	10 (4.9)	6 (6.1)	4 (3.7)	
	Correct	189 (91.7)	87 (87.9)	102 (95.3)	
Performs mathematical calculation (*n* = 794)	No	1 (0.1)	1 (0.3)	0	< .001*
	Partially	19 (2.4)	17 (4.9)	2 (0.4)	
	Incorrectly	62 (7.8)	58 (16.7)	4 (0.9)	
	Correctly	712 (89.7)	271 (78.1)	441 (98.7)	
Contextualization to the case (*n* = 328)	No	26 (7.9)	19 (10.4)	7 (4.8)	< .001
	Partial	27 (8.2)	3 (1.6)	24 (16.4)	
	Incorrect	83 (25.3)	73 (40.1)	10 (6,8)	
	Correct	192 (58.5)	87 (47.8)	105 (71.9)	
Checks that the result is realistic (*n* = 343)	No	28 (8.1)	19 (10.7)	9 (5.4)	< .001
	Partially	21 (6.1)	12 (6.8)	9 (5.4)	
	Incorrectly	112 (32.9)	92 (52.0)	21 (12.6)	
	Correctly	182 (52.9)	54 (30.5)	128 (76.6)	
Extracts the key information from the question (*n* = 793)	No	43 (5.4)	38 (11.0)	5 (1.1)	< .001
	Partially	348 (43.9)	173 (49.9)	175 (39.2)	
	Incorrectly	16 (2.0)	16 (4.6)	0	
	Correctly	386 (48.7)	120 (34.6)	266 (59.6)	
Understands the question (*n* = 863)	Partially	61 (7.1)	59 (14.5)	2 (0.4)	< .001
	Incorrectly	146 (16.9)	145 (35.5)	1 (0.2)	
	Correctly	656 (76.0)	204 (50.0)	452 (99.3)	
Units of measurement in their answer (*n* = 794)	No	27 (3.4)	20 (5.8)	7 (1.6)	< .001
	Partial	29 (3.7)	20 (5.8)	9 (2.0)	
	Incorrect	1 (0.1)	1 (0.3)	0	
	Correct	737 (92.8)	306 (88.2)	431 (96.4)	
Correct units of measurement in their answer (*n* = 772)	Partial	37 (4.8)	29 (8.8)	8 (1.8)	< .001
	Incorrect	83 (10.8)	79 (23.9)	4 (0.9)	
	Correct	652 (84.5)	223 (67.4)	429 (97.3)	
Appropriate volume of diluent (*n* = 251)	Incorrect	60 (23.9)	55 (35.3)	5 (5.3)	< .001
	Correct	191 (76.1)	101 (64.7)	90 (94.7)	
Use of conversion factor (*n* = 168)	Incorrect	8 (4.8)	8 (11.0)	0	.001
	Correct	160 (95.2)	65 (89.0)	95 (100)	
Use of the ratio-proportion method (*n* = 554)	Incorrect	60 (10.8)	57 (25.8)	3 (0.9)	< .001
	Correct	494 (89.2)	164 (74.2)	330 (99.1)	
Understanding of unit equivalences (*n* = 289)	Incorrect	109 (37.7)	107 (56.9)	2 (2.0)	< .001
	Correct	180 (62.3)	81 (43.1)	99 (98.0)	
Understanding of percentages (*n* = 122)	Incorrect	63 (51.6)	59 (89.4)	4 (7.1)	< .001
	Correct	59 (48.4)	7 (10.6)	52 (92.9)	
Understanding of maximum concentration and minimum dilution (*n* = 152)	Incorrect	74 (48.7)	71 (67.0)	3 (6.5)	< .001
	Correct	78 (51.3)	35 (33.0)	43 (93.5)	
Understanding of continuous and intermittent infusion (*n* = 436)	Incorrect	87 (20.0)	83 (31.7)	4 (2.3)	< .001
	Correct	349 (80.0)	179 (68.3)	170 (97.7)	
Infusion rate calculation (*n* = 345)	Incorrect	130 (37.7)	122 (56.0)	8 (6.3)	< .001
	Correct	215 (62.3)	96 (44.0)	119 (93.7)	
Infusion time calculation (*n* = 355)	Incorrect	105 (29.6)	100 (45.5)	5 (3.7)	< .001
	Correct	250 (70.4)	120 (54.5)	130 (96.3)	
Knowledge of equipment for drug administration (*n* = 232)	Incorrect	65 (28.0)	59 (36.6)	6 (8.5)	< .001
	Correct	167 (72.0)	102 (63.4)	65 (91.5)	
Follows the correct steps (*n* = 794)	No	260 (32.7)	253 (72.9)	7 (1.6)	< .001
	Yes	534 (67.3)	94 (27.1)	440 (98.4)	
Error carried forward (*n* = 347)	No	78 (22.5)	77 (22.3)	1 (50.0)	.400
	Yes	269 (77.5)	268 (77.7)	1 (50.0)	

The total n for the categories considered in the left-hand column varies because they were not all relevant to every exercise; their relevance depended on the question being asked. *Fisher’s exact test; for this comparison we grouped the data for the rating categories No/Partial/Incorrect

Importantly, students also had difficulties with more basic aspects such as calculating the IV infusion rate (37.7%), the infusion time (29.6%), and the volume of solution in which the drug should be dissolved (23.9%). Furthermore, in 51.3% of cases, students did not correctly extract the key information from the question when trying to solve the problem, and this was associated with significantly more incorrect answers (*p* < .001). Finally, we found that in 16.9% of the exercises analyzed, the student had clearly not understood the task; this was more commonly the case with more difficult exercises or calculations involving more than one step.

Comparison of exercises across the three course years showed that the overall number of errors tended to decrease as students progressed through the degree program, except in relation to performing a mathematical calculation (*p* = .997), for which the proportion of exercises containing an error remained fairly stable (between 5 and 9%). However, the proportion of students who failed to understand the question increased across course years as the exercises set became more complex (year 1: 5.7%; year 2: 24%; year 3: 37.7%; *p* < .001), with a similar trend being observed for the percentage of students who did not follow the correct steps in solving the problem (year 1: 35.2%; year 2: 29.3%; year 3: 52.6%; *p* = .043).

### Answers according to type of exercise

#### Unit equivalences

The unit equivalences (theory) exercise in year 2 comprises five questions, and only 8 (11.6%) students answered them all correctly. The unit equivalence they were most familiar with was *g – mg* (84%), followed by *ml – cc, ml – microdrops* (both 65.2%), *mcg (μg) – mg* (52.2%), and, finally, the percentage equivalence, *% = mg/ml* (18%).

#### Exercises referring to an adult patient

##### Unit conversion

Consistent with the overall results for the unit equivalences (theory) test, we found that 40.6% of students in year 2 made a mistake when converting units in the clinical exercises. The evaluators commented that two students obtained an incorrect answer because they took the abbreviation *mcg* to mean *microdrops* (it should be noted here that the Spanish word for drop is *gota*, which led these students to misinterpret the letter *g* in *mcg*). Another comment made was: “they get a wrong answer because they don’t know how to convert *mg* to *mcg*”. Notably, one student failed to double-check an answer that, in practical terms, was completely unrealistic (dose to administer of 10^−6^ mg).

##### Drug concentration calculations

In the dose calculation exercise involving a percentage (year 2), 90.6% of students obtained a correct answer, much higher than the proportion who, in the theory test, knew that % = mg/ml. It should be noted that % was defined in this practical exercise.

When asked to calculate the drug concentration over time (exercise above the knowledge level expected of year 2 students), 25.6% of students did not calculate the infusion time, 20.9% did not understand the question, and 27.9% made an error that they then carried forward in their calculation. In their comments, the evaluators noted that on six occasions the final answer was incorrect due to rounding or use of a recurring decimal. Other comments of note included: “treats the diluent as part of the drug dose”, “doesn’t apply the rule-of-three method correctly”, and “calculation is correct, but understands minutes instead of hours”.

One of the exercises in year 3 asked students to maintain a prescribed dose for a different drug concentration and infusion rate. The most common errors here were a failure to understand the question (48%) and not knowing how to calculate the infusion rate (50%). In their comments, evaluators noted that 13 students did not how to apply the rule-of-three method, and four did not correctly extract the information from the question, leading to wrong answers.

##### Basic dose calculation and administration rate

In the clinical case exercise in year 1, students had to calculate both the dose of a prescribed IV drug from the stock available and also the corresponding infusion rate. It can be seen in Table 5 that only 36% of students answered both parts of this exercise correctly, and in most cases this was due to difficulty calculating the infusion rate (*p* < .001).

**Table 5:** Results for the clinical case calculation in year 1

n (%)		Dose calculation		
		Correct	Incorrect	Total
Infusion rate	Correct	27 (36.0)	4 (5.3)	31 (41.3)
	Incorrect	25 (33.3)	19 (25.3)	44 (58.7)
	Total	52 (69.3)	23 (30.7)	75 (100)

The proportion of year 2 students who correctly calculated the dose of an IV drug was higher than in year 1 (correct: 69.3% in year 1 vs. 84.2% in year 2), although they continued to struggle with the calculation of infusion rate (correct: 41.3% in year 1 vs. 14.1% in year 2).

Regarding the comments made by evaluators about students’ dose calculations, it was noted that six students in year 1 “were confused about the drug stock and thought it was a powder vial rather than a liquid ampoule”. Examples of comments made about the year 2 exercises included: “gets a wrong answer due to rounding in a previous calculation”, “gives the wrong units”, and “makes a mistake when multiplying”. With respect to the infusion rate calculations, comments related to the year 1 exercise included “mistakes intermittent infusion for continuous infusion”, “forgets to add the drug volume to the total amount of IV solution”, and “chooses the wrong kind of IV solution”, while those for year 2 exercises included “performs the calculation in ml/h instead of drops/minute”, “uses the wrong IV infusion set”, and “doesn’t know how to convert units”.

#### Exercises referring to a pediatric patient

The first three exercises analyzed here come from year 2 of the degree program, while the clinical case exercises correspond to year 3 students. Only the first of the 3 year 2 exercises (Dose calculation by patient’s weight) corresponded to the knowledge level expected of year 2 students.

##### Dose calculation by patient’s weight

The majority of students were able to perform this calculation correctly. Comments made by evaluators regarding incorrect answers included: “divides instead of multiplies so gets it wrong”, “doesn’t know how to do the calculation”, and “wrong units”.

##### Total infusion time, taking into account the maximum flow rate

The large majority of errors here were due to a lack of understanding of the concepts maximum concentration and minimum dilution (52.8%), and to confusion between intermittent and continuous infusion (54.7%). Overall, 56.6% of students were unable to calculate the total infusion time.

##### Volume of diluent, taking into account the maximum concentration

Only two students (5.3%) correctly answered this exercise. Most of the errors were related to not understanding the concepts maximum concentration or minimum dilution (94.7%) and to not knowing the appropriate volume of diluent that should be used (73.7%). In addition to noting that students didn’t know how to perform the calculation, evaluators also commented that many of them only calculated the drug dose (“only calculates the ml of aciclovir”) and failed to calculate the corresponding volume of diluent.

##### Clinical case exercises

We analyzed two clinical pediatric exercises from year 3, both of which required students to make calculations involving more than one step. One of these cases comprises two parts, which were analyzed separately.

The most frequent errors were not contextualizing their answer to the case (between 24.6 and 73.3% of answers, depending on the exercise), not checking that the result was realistic and made sense (between 31.2 and 65.6%), and not fully understanding the question (between 7.8 and 26.2%). It should be noted that in one of the exercises, 14.1% only partially completed the mathematical calculation and thus could not obtain a final result. In the other two exercises, some students (6.3 and 14.8%, respectively) did not know how to calculate the appropriate amount of diluent. Finally, and related to the fact that these exercises involved multi-step calculations, we found that although the correct steps were followed by between 52.5 and 100% of students (depending on the exercise), the final result was incorrect in between 42.1% of 63.9% of cases due to an error being carried forward.

## Discussion

This article presents a detailed analysis of the errors made by nursing undergraduates when performing written dose calculation exercises. The results add to existing evidence regarding the kinds of problems that nursing students have with calculation exercises [21]. Our analysis suggests that students’ overall level of calculation skills is limited, although they are generally able to perform basic dose calculations. The proportion of students who correctly answered the exercises set was slightly below that reported in some studies [19, 23, 24], but similar to that observed by Bagnasco et al. [4]. However, our results should be interpreted with caution as a small number of the year 2 exercises we analyzed included questions that went beyond the level of knowledge that students were expected to have at this point in their studies; as noted above in Method (sub-section *Examination Papers Reviewed*), these more difficult problems were deliberately included as a way of enabling us to track the progress of individual students in subsequent years of the degree program. If we omit these three questions from the analysis, the mean grade obtained by students on this exam increases from 5.2 (SD 2.2) to 6.3 (SD 2.5) out of 10, a figure consistent with pass rates reported in the aforementioned literature. Given that these year 2 students also have two more years of their degree ahead of them, it would obviously be interesting to follow them up and compare their level of achievement on the same kinds of dose calculation problems in years 3 and 4, thus providing an indication of the level they have reached by the time they transition to professional practice.

One of the errors we observed, which was also discussed by Bagnasco et al. [4], reflected students’ difficulty with converting units. In Spain, this concept is taught during secondary education (with the exception of *% = mg/ml* and *drops – ml*), and hence students should be able to perform this operation by the time they enter university. More specifically, we found that students had greater difficulty moving up the scale of units (e.g., from *mcg* to *mg*) rather than down, and also that some students correctly converted units in the clinical case exercise but not in the unit equivalences theory test, and vice-versa. Given their importance in clinical practice, greater attention needs to be paid to these concepts during students’ training.

Our analysis also showed that students were more likely to produce a correct answer when they applied a structured approach to the problem (e.g., extract all the key information from the question, use the correct units, and follow the correct calculation steps). This structured approach is taught at earlier stages of education in our country, although it may not be adequately assimilated by all students. If this is the case, then it could have a negative impact on their ability to perform medication calculations, which tend to be more complex in content than the purely arithmetic problems they will have been set during secondary education [21, 25].

Obviously, students would most likely find calculation exercises easier if they involved a single task expressed in clear and concise terms. However, this would not reflect clinical reality, because in practice a dose calculation is made in the context of a specific patient and other variables that may affect the final result must also be taken into account. Setting students contextualized case exercises therefore helps to reduce the theory-practice gap [26]. Accordingly, the exercises analyzed in this study involved clinical scenarios of varying complexity, and it was noticeable that the easier questions (those with just one or two calculation steps) were more likely to be answered correctly than were the more complex multi-step problems. This is reflected in the proportion of exercises where the student had clearly not understood the question (16.9%), which tended to be the more difficult problems involving more than one calculation [25]. In those exercises (*n* = 328) where it was possible to analyze whether the student had contextualized their result and checked whether it was realistic and made sense, we found that 45.5% failed to do so. In order to solve problems of this kind, students must understand precisely what they are being asked to do and extract from the question the key information they need to perform the calculation correctly [21]. In this context, Grunetti et al. [19] found that while students may find it helpful to use a calculator, this can also produce a false sense of security, such that they do not then consider whether the result makes sense or not. This kind of error is much more common among students who do not have a good grasp of the principles of mathematical calculation or who struggle with logical reasoning [27]. An example from our analysis was a student who did not question a final result giving a drug dose of 10^− 6^ mg, even though it should be obvious that it is impossible to administer such a small amount to a patient. In our view, clinical simulation with manikins is a highly useful tool for helping students develop their skills in this respect [27, 28], insofar as it allows them to see the practical result of their calculations (i.e., the drug volume to administer), in addition to providing them with an opportunity to improve their critical thinking [29] and to learn from mistakes and their peers [30]. It should also be noted that simulation is not a stress-free experience for students, as they will be observed and be set a time limit for performing the task, and in this respect it more closely resembles the realities of clinical practice [31]. As an alternative or complement to simulation, one might also use more active learning strategies or those in which students can see the material they need or visualize what is being explained to them, rather than it being presented in abstract or purely theoretical terms [1, 26].

Another important difficulty that students had, regardless of the course year they were in, concerned calculation of the IV infusion rate. In our view, this suggests a gap between theory and practice in this respect, because although students are taught in class how to calculate the infusion rate, they are unlikely to see clinical nurses calculate the drip rate in drops/minute as this is usually estimated by the nurse when setting up a manual IV set [32]. By contrast, students do gain practical experience of calculating the rate in ml/h, because these are the units used with infusion pumps. In accordance with Hedlund et al. [3], we also found that students had difficulties in calculations involving the total administration time or the volume of diluent, both of which are more theoretical pharmacological concepts. A task for future research would be to examine in more detail the possible relationship between students’ exposure to these kinds of calculations while on clinical placement and their performance in written examinations.

The present study has several limitations that derive from the retrospective design and the fact that the analysis is based solely on written dose calculation exercises. One is that we do not know why some exercises were left blank, that is to say, whether it was because the student did not know how to solve the problem or simply ran out of time during the exam. Neither is it clear whether the number of correct answers would have been greater if students had been allowed more time or did not feel the pressure of an exam situation. On a related issue, we have no way of knowing whether those students who gave a correct answer were confident about their calculations or got there more by luck than judgment. It would therefore be useful in future studies to complement an analysis of this kind with qualitative feedback from students themselves regarding the difficulties they experienced and their level of confidence in their answers. Our approach here also provides no insight into students’ thought process or reasoning, which would be necessary in order to understand more about why precisely they made the errors they did. This would also be an interesting topic for future research. As noted earlier, three of the exercises set during year 2 imply a knowledge level above that expected of students at this stage of their training, the rationale being that this allows us to track the progress of individual students over subsequent years of their studies. We acknowledge, however, that in the context of the present analysis the inclusion of these exercises may bias the results obtained for year 2 students, which must therefore be interpreted with caution. A final limitation to consider is that we have no comparative data among the years of nursing degree regarding the dose calculation skills. Moreover, students spend year 4 almost entirely on placement, making it difficult to schedule a classroom-based test their ability in this respect. In order to build on the present results, it would be useful to introduce a final written examination (such as that used with year 2 students) at the end of every year so as to explore the evolution of the knowledge and level that our students reach by the time they graduate.

Notwithstanding these limitations, our study also has two important strengths: One is the large number and variety of dose calculation exercises analyzed, while the other is the detailed analysis of the aspects that students find most difficult.

## Conclusions

Nursing students have adequate skills when it comes to basic dose calculations, but struggle with more complex problems, although they tend to improve as they progress through their studies. The most common errors we observed were related to not understanding or not extracting the key information from the question, not following the correct steps in their calculations, and a lack of basic knowledge such as how to convert units. The fact that some students did not consider whether their answer was realistic and made sense in a clinical context is problematic from the point of view of safe medication management. The use of high-fidelity simulation scenarios during their training could play an important role in helping them improve their skills in this respect.

## Relevance to clinical practice

In order to ensure that nursing students are proficient in medication calculation by the time they graduate, nurse educators need to identify the specific aspects and steps in the process that students find most difficult, thus enabling instruction to be targeted where it is most needed. The present analysis of students’ answers to a series of dose calculation exercises of varying levels of complexity shows that their errors cannot be attributed solely to poor calculation skills, insofar as they involved different aspects and stages of the problem-solving process. Notably, students often struggled to understand key concepts associated with dose calculation and failed to follow the correct steps when performing the exercises set. Nurse education programs must, in addition to developing students’ mathematical competence, ensure they acquire an adequate understanding of the key concepts (both numerical and clinical) underpinning medication calculation, as well as an appreciation of the importance of checking that their result is realistic and makes sense in relation to each individual patient.

## Supplementary Information

**Additional file 1.**

## Data Availability

The regulations of our university that cover the use of data from students do not allow us to share these datasets publicly. However, data in the form of aggregated results are available from the corresponding author upon reasonable request.
