# Supplementary material for: Where do nursing students make mistakes when calculating drug doses? A retrospective study
Source: BMC Nurs. 2022 Nov 10;21:309. doi: 10.1186/s12912-022-01085-9 (PMC9648043; doi:10.1186/s12912-022-01085-9)
Supplement: Supplementary file 1 — Additional file 1. [file 12912_2022_1085_MOESM1_ESM.docx]

**SUPPLEMENTARY MATERIAL**

**Supplementary material 1**

**Examples of each type of exercise**

| Basic dose calculation | You have to administer 3 mg of ambroxol (Mucosolvan) to a patient through a nasogastric tube. The stock available contains 0.3 mg/ml in liquid form. How many ml should you administer? |
| --- | --- |
| Unit conversion | Mary has been admitted to the surgical ward and has to undergo a painful procedure. The doctor has prescribed an IV bolus of fentanyl 0.075 mg. Stock ampoules contain fentanyl 150 mcg/3 ml in liquid form. How many ml should you administer to the patient? |
| Infusion rate | You have a patient who is nil by mouth prior to surgery and the doctor has ordered a saline drip (450 ml NaCL 0.9% for 8 h). Calculate the drip rate you should set the macroset to deliver. |
| Total infusion time, taking into account the maximum flow rate | A transplant patient requires prophylaxis for Candida infection. The prescription is fluconazole 83 mg/12 h. Calculate the total infusion time if the maximum flow rate of the system is 200 mg/h. |
| Dose calculation involving a percentage | You have to administer IV heparin sodium. The stock available is 5% heparin sodium (1 mg = 50 mg) in vials of 25000 IU in 5 ml of liquid. The patient requires a bolus of 7500 IU. How many ml do you need given the stock available? |
| Dose according to patient's weight | You have to administer IV methylprednisolone to a child aged 18 months and weighing 11 kg. The prescription is 1.5 mg/kg/day. What dose should you administer every 12 h? |
| Drug concentration | A patient is receiving a continuous infusion 10 ml/h of noradrenaline. The infusion bag contains 40 mg in 250 ml of 5% dextrose solution. How many mg/min are you administering to the patient? |
| Volume of diluent, taking into account the maximum concentration | It is decided to start antiviral prophylaxis in a patient with neutropenia. The prescription is aciclovir 175 mg/8 h IV (stock ampoules contain 250 mg/10 ml in liquid form). Calculate how many ml of aciclovir you need for the prescribed dose and what volume of diluent you should use if the maximum concentration you should infuse is 7 mg/ml. |

**Supplementary material 2**

**Dose calculation questionnaire used in Year 2**

1. Indicate the following unit equivalences:
   1. 1ml =_____cc
   2. 1 mcg (µg) =_____mg
   3. 1 g =______mg
   4. 1 ml =_____microdrops
   5. 1% = ____mg/____ml
2. You have to administer 3 mg of Mucosolvan to a patient through a nasogastric tube. The stock available contains 0.3 mg/ml in liquid form. How many ml should you administer?
3. Paula (aged 24 years) presents at the emergency room with dyspnea. She has a history of asthma and the duty physician prescribes a bronchodilator and steroids. The steroid prescription is methylprednisolone 40 mg IV. The stock available comes in the form of two ampoules, one containing 15 mg of methylprednisolone in powder form, the other containing 2 ml of water in which to dissolve the powder for injection. How many ml of dissolved drug should you administer to the patient?
4. Mary has been admitted to the surgical ward and has to undergo a painful procedure. The doctor has prescribed an IV bolus of fentanyl 0.075 mg. Stock ampoules contain fentanyl 150 mcg/3 ml in liquid form. How many ml should you administer to the patient?
5. A child who has received a hematopoietic stem cell transplant requires prophylaxis for Candida infection. The prescription is fluconazole (Diflucan®) 83 mg/12 h IV. Calculate the total infusion time if the maximum flow rate of the system is 200 mg/h.
6. You have to administer IV heparin sodium. The stock available is 5% heparin sodium (1 mg = 50 mg) in vials of 25000 IU in 5 ml of liquid. The patient requires a bolus of 7500 IU. How many ml do you need given the stock available?
7. You have to administer IV methylprednisolone to a child aged 18 months and weighing 11 kg. The prescription is 1.5 mg/kg/day. What dose should you administer every 12 h?
8. A patient is receiving a continuous infusion 10 ml/h of noradrenaline. The infusion bag contains 40 mg of noradrenaline in 250 ml of 5% dextrose solution. How many mg/min are you administering to the patient?
9. It is decided to start antiviral prophylaxis in a pediatric patient with neutropenia. The prescription is aciclovir 175 mg/8 h IV (stock ampoules contain 250 mg/10 ml in liquid form). Calculate how many ml of aciclovir you need for the prescribed dose and what volume of diluent you should use if the maximum concentration you should infuse is 7 mg/ml.
10. You have a patient who is nil by mouth prior to surgery and the doctor has ordered a saline drip (450 ml NaCL 0.9% for 8 h). Calculate the drip rate you should set the macroset to deliver.

**Supplementary material 3**

**Definition of the 23 categories used in analyzing the exercises**

| 1 | Uses information given about the stock available | Correctly interprets the information given about the stock available. For example: Ampoules containing 2 mg/ml in liquid form. |
| --- | --- | --- |
| 2 | Consideration of the diluent solution | Correctly considers the diluent solution when doing the exercise. For example, if the drug has to be dissolved in 5% dextrose solution, the student does not consider the 5% as an element to include in the calculation. |
| 3 | Performs mathematical calculation | Performs the pure mathematical calculation correctly, even if the information it contains is incorrect. |
| 4 | Contextualization to the case | Takes into account the case described in the exercise. For example, with a pediatric patient the student does not dissolve a powder in 100 ml of liquid, but uses a smaller volume. |
| 5 | Checks that the result is realistic | Checks that the result makes sense. For example: If their answer is a recurring decimal they round it up or down so that it is realistic in a clinical context; if the result suggests they need 10 powder sachets, they show an awareness that it is incorrect. |
| 6 | Extracts the key information from the question | Extracts and uses the key information from the question when solving the exercise. |
| 7 | Understands the question | Understands what they are being asked to do. |
| 8 | Units of measurement in their answer | Includes the units in their numerical answer to the problem. |
| 9 | Correct units of measurement in their answer | Uses the correct units in their numerical answer to the problem. |
| 10 | Appropriate dilution | The dilution considered by the student when solving the problem is appropriate for the patient described in the question. |
| 11 | Method of solving the problem | Method used by the student to solve the problem. Two methods were considered: ratio-proportion and conversion factor. |
| 12 | Use of the problem-solving method chosen | The student knows how to apply the method chosen to solve the problem. |
| 13 | Understanding of unit equivalences | Understands the unit equivalences required to solve the problem. For example: mg to mcg, or g to mg |
| 14 | Understanding of percentages | Understands the percentage equivalents needed to solve the problem. |
| 15 | Understanding of maximum concentration and minimum dilution | Understands the concepts of maximum concentration and minimum dilution when these are needed to solve the problem. |
| 16 | Understanding of continuous and intermittent infusion | Understands the difference between an intermittent IV infusion (e.g., for 30 min every 8 h) and a continuous infusion (e.g., for 8 h). |
| 17 | Knowledge of equipment for drug administration | Takes into account the type of equipment mentioned in the question when solving the problem. For example, if the question says the drug is to be administered using a macroset, the student calculates the infusion rate in drops/minute. |
| 18 | Error carried forward | The student makes a mistake that leads to error in subsequent steps of the calculation. This aspect was not evaluated in exercises where the final answer was correct. |
| 19 | Infusion rate calculation | Is able to calculate the IV flow rate. |
| 20 | Infusion time calculation | Is able to calculate the infusion time for the drug described in the question. |
| 21 | Follows the correct steps | Follows the correct steps in solving the problem. |
| 22 | Completes the exercise | Clearly presents a final answer to their calculation (usually in a box or by indicating that it is the solution to the problem). |
| 23 | Correct result | The student obtains a correct result from the calculation. |
